# Effect of guanylin peptides on pancreas steatosis and function in experimental diet-induced obesity and after bariatric surgery

**DOI:** 10.3389/fendo.2023.1185456

**Published:** 2023-05-18

**Authors:** Aarón Otero, Sara Becerril, Marina Martín, Javier A. Cienfuegos, Víctor Valentí, Rafael Moncada, Victoria Catalán, Javier Gómez-Ambrosi, María A. Burrell, Gema Frühbeck, Amaia Rodríguez

**Affiliations:** ^1^ Metabolic Research Laboratory, Clínica Universidad de Navarra, Pamplona, Spain; ^2^ CIBER Fisiopatología de la Obesidad y Nutrición (CIBEROBN), Instituto de Salud Carlos III, Madrid, Spain; ^3^ Obesity and Adipobiology Group, Instituto de Investigación Sanitaria de Navarra (IdiSNA), Pamplona, Spain; ^4^ Department of Pathology, Anatomy and Physiology, University of Navarra, Pamplona, Spain; ^5^ Department of Surgery, Clínica Universidad de Navarra, Pamplona, Spain; ^6^ Department of Anesthesia, Clínica Universidad de Navarra, Pamplona, Spain; ^7^ Department of Endocrinology and Nutrition, Clínica Universidad de Navarra, Pamplona, Spain

**Keywords:** uroguanylin, guanylin, guanylate cyclase 2C, β-cell function, lipogenesis

## Abstract

**Introduction:**

Obesity contributes to ectopic fat deposition in non-adipose organs, including the pancreas. Pancreas steatosis associates with inflammation and β-cell dysfunction, contributing to the onset of insulin resistance and type 2 diabetes. An improvement of pancreatic steatosis and indices of insulin resistance is observed following bariatric surgery, but the underlying mechanisms remain unknown. We sought to analyze whether guanylin (GUCA2A) and uroguanylin (GUCA2B), two gut hormones involved in the regulation of satiety, food preference and adiposity, are involved in the amelioration of pancreas fat accumulation after bariatric surgery.

**Methods:**

Pancreas steatosis, inflammation, islet number and area were measured in male Wistar rats with diet-induced obesity (n=125) subjected to surgical (sham operation and sleeve gastrectomy) or dietary (pair-fed to the amount of food eaten by gastrectomized animals) interventions. The tissue distribution of guanylate cyclase C (GUCY2C) and the expression of the guanylin system were evaluated in rat pancreata by real-time PCR, Western-blot and immunohistochemistry. The effect of guanylin and uroguanylin on factors involved in insulin secretion and lipogenesis was determined *in vitro* in RIN-m5F β-cells exposed to lipotoxic conditions.

**Results:**

Sleeve gastrectomy reduced pancreas steatosis and inflammation and improved insulin sensitivity and synthesis. An upregulation of GUCA2A and GUCY2C, but not GUCA2B, was observed in pancreata from rats with diet-induced obesity one month after sleeve gastrectomy. Interestingly, both guanylin and uroguanylin diminished the lipotoxicity in palmitate-treated RIN-m5F β-cells, evidenced by lower steatosis and downregulated lipogenic factors *Srebf1, Mogat2* and *Dgat1*. Both guanylin peptides reduced insulin synthesis (*Ins1* and *Ins2*) and release from RIN-m5F β-cells, but only guanylin upregulated *Wnt4*, a factor that controls β-cell proliferation and function.

**Discussion:**

Together, sleeve gastrectomy reduced pancreatic steatosis and improved β-cell function. Several mechanisms, including the modulation of inflammation and lipogenesis as well as the upregulation of GUCA2A in the pancreas, might explain this beneficial effect of bariatric surgery.

## 1 Introduction

Obesity is a major health problem worldwide due to its rising prevalence and its association with several serious diseases, including type 2 diabetes, cardiovascular disease and cancer (1, 2). Moreover, obesity causes the accumulation of fat in non-adipose organs, such as the liver (non-alcoholic fatty liver disease [NAFLD] or non-alcoholic steatohepatitis [NASH]), muscle, heart and pancreas, leading to deleterious effects on systemic metabolism (3, 4). Pancreatic steatosis was described for the first time in 1933 by Ogilvie (5) in a *post-mortem* study showing an increased pancreatic fat in 19 cases of obesity compared with 19 control cases (17% *vs* 9%). Several synonyms are reported in the literature for the pancreatic steatosis according to different clinical pathologies, such as pancreatic fat accumulation, non-alcoholic fatty pancreas (NAFP), pancreatic lipomatosis, fatty replacement or fatty infiltration of the pancreas (6, 7). The death of acinar cells induced by hematochromatosis, viral infection or duct obstruction, leads to an irreversible infiltration of fat, known as pancreatic fatty replacement. By contrast, in pancreatic steatosis or NAFP, obesity leads to a potentially reversible pancreatic fat accumulation. Growing body of evidence supports that NAFP is associated with lipotoxicity, insulin resistance and inflammation, leading to impaired β-cell function and glucose metabolism (8, 9). Nonetheless, the pathophysiology of NAFP remains unclear. It has been proposed that short-term exposure of β-cells to free fatty acids (FFA) increases glucose-stimulated insulin secretion, while obesity-associated chronic exposure to high FFA levels promotes β-cell hypertrophy and insulin hypersecretion, ultimately causing β-cell dysfunction and death (10–14).

The intestinal peptides guanylin and uroguanylin are the ligands for the transmembrane receptor guanylate cyclase C (GUCY2C), and participate in several physiological functions, including the regulation of intestinal fluid homeostasis, natriuresis, diuresis and maintenance of intestinal barrier integrity and inflammatory responses (15–17). In addition, guanylin and uroguanylin are secreted as prohormones after nutrient ingestion, playing an important role in obesity as satiety factors (18, 19). Proguanylin (GUCA2A) and prouroguanylin (GUCA2B) are enzymatically cleaved, and active peptides can activate hypothalamic and midbrain circuits that decrease appetite upon binding GUCY2C (19–21). Uroguanylin also increases Ca^2+^ concentrations in astrocytes *via* GUCY2C-dependent signalling pathway modulating neuronal activity in cerebral and cerebellar cortex (22, 23). Guanylin peptides are involved in the regulation of food preferences (24, 25), adiposity, and energy expenditure (26–28) *via* central and peripheral mechanisms. Intestinal and circulating GUCA2B are decreased in experimental (19, 20, 29, 30) and human (25, 28, 31, 32) obesity, resulting in an impaired uroguanylin-GUCY2C endocrine axis in the obese state. A blunted endocrine secretion of GUCA2B and/or decreased intestinal expression of guanylin peptides and its receptor GUCY2C has been also observed in patients with ulcerative colitis (33), chronic constipation (34) and colorectal cancer (35). In the recent years, targeting the uroguanylin-GUCY2C endocrine axis using peptides (36) or drugs (26) has been proposed as a therapeutic strategy for the treatment of obesity (18).

We herein hypothesized that the guanylin system might be involved in pancreatic steatosis due to its anti-obesity properties. The pancreas represents a production site and target of guanylin peptides (37, 38). In the exocrine pancreas, guanylin regulates electrolyte/fluid secretion in ductal cells (37), while uroguanylin exerts anti-proliferative actions in pancreatic ductal adenocarcinoma cell lines (39). However, controversial reports regarding the function of guanylin peptides in the regulation of insulin secretion in the endocrine pancreas are found in the literature (40, 41). Therefore, we first explored the impact of weight gain induced by diet-induced obesity and weight loss achieved by bariatric surgery in pancreas expression of guanylin peptides and their receptor GUCY2C in rats. The potential association of changes in the expression of the guanylin system with key regulatory molecules of pancreas steatosis, inflammation and β-cell function was examined. Finally, we analyzed *in vitro* the direct effect of guanylin and uroguanylin on insulin secretion and lipogenesis in RIN-m5F β-cell line under lipotoxic conditions.

## 2 Material and methods

### 2.1 Experimental animals and study design

Four-week-old male Wistar rats (n=125) supplied by the breeding house of the University of Navarra were housed individually in a specific pathogen-free facility with controlled room temperature (20-22 °C), relative humidity (50 ± 10%) and on a 12-h light/dark cycle (08:00-20:00 lights on). Animals were fed *ad libitum* during 4 months with either a normal diet (ND) (n=25) (12.1 kJ: 4% fat, 82% carbohydrate and 14% protein, diet 2014S, Harlan, Teklad Global Diets, Harlan Laboratories Inc., Barcelona, Spain) or a high-fat diet (HFD) (n=100) (23.0 kJ/g: 59% fat, 27% carbohydrate and 14% protein, diet F3282; Bio-Serv, Frenchtown, NJ, USA). Body weight and food intake were registered weekly and a group of rats (n=24) was maintained on a HFD to monitor the effect of obesity. The rest of the rats with diet-induced obesity were randomly assigned to 3 weight-matched groups: i) sleeve gastrectomy (resecting 60% of total gastric volume) (n=26); ii) sham surgery without gastric resection (n=27); and iii) pair-feeding that received the same amount of food eaten by the group with gastrectomy (n=23). Anesthesia, sham surgery and sleeve gastrectomy were performed according to previously described methodology (13). After surgery, animals were kept on a liquid diet with 5% glucose and 0.9% saline solution for 3 days and, thereafter, rats were fed a ND. Two weeks after surgery, oral glucose tolerance (OGTT) and intraperitoneal insulin tolerance (IPITT) tests were performed after an overnight fast (12 h). Blood glucose concentrations were measured at 0, 15, 30, 60, 90 and 120 min after the oral glucose challenge (2 g/kg of body weight) or intraperitoneal insulin administration (0.15 IU/mL) with an automatic glucose sensor (Ascensia Elite, Bayer, Barcelona, Spain), and areas under the curve (AUC) were determined. Four weeks after the surgical and dietary interventions, rats were sacrificed by decapitation after an 8-h fasting period. All experimental procedures conformed to the European Guidelines for the care and use of Laboratory Animals (directive 2010/63/EU) and were approved by the Ethical Committee for Animal Experimentation of the University of Navarra (049/10).

### 2.2 Blood assays

Blood samples were centrifuged at 700 *g* at 4°C for 15 min to obtain sera samples. Serum glucose was determined by an automatic glucose sensor (Ascencia Elite, Barcelona, Spain). Serum leptin and insulin (#90030 and #90060, Crystal Chem, Inc., Chicago, IL, USA) were determined by ELISA (42, 43). Intra- and inter-assay coefficients of variation were 5.4% and 6.9% for leptin, and 3.5% and 6.3% for insulin. Insulin resistance was calculated using the homeostasis model assessment (HOMA-IR), calculated with the following formula: fasting insulin (µU/mL) × fasting glucose (mmol/L)/22.5). Plasma concentrations of GUCA2A (CSBEL010047RA, Cusabio Biotech., Wuhan, China), GUCA2B (CSBEL010048RA, Cusabio) and GLP-1 (EZGLP1T-36K, Millipore and 81507, Crystal Chem, Chicago, IL, USA) were measured using ELISA kits following the manufacturer’s protocols with intra- and inter-assay coefficients of variation being <10% and <12%, respectively.

### 2.3 Pancreas handling

Pancreas was carefully dissected out, weighed, snap-frozen and stored at -80 °C until RNA and protein extraction. A small portion of pancreas was fixed in 4% formaldehyde for histological analyses. Total RNA isolation and purification from pancreas (50 mg) and RIN-5mF β-cells was performed using TRIzol^®^ Reagent (Invitrogen, Carlsbad, CA) and RNeasy Mini Kit (Qiagen, Hilden, Germany), according to the manufacturer’s instructions. Total proteins from pancreas (50 mg) were extracted using RIPA buffer (0.1% SDS, 1% Triton X-100, 5 mmol/L EDTA·2H_2_O, 1 mol/L Tris, 150 mmol/L NaCl, 1% sodium deoxycholate, pH 7.40) supplemented with a protease inhibitor cocktail (CompleteTM Mini-EDTA free, Roche, Mannheim, Germany). Samples were centrifuged at 16,000 *g* at 4 °C for 15 min and total protein concentrations were quantified by the Bradford assay (Bio-Rad Laboratories, Inc., Hercules, CA, USA) using bovine serum albumin (BSA) (Sigma, Darmstadt, Germany) as standard.

### 2.4 RIN-m5F β-cell culture and treatment

RIN-m5F rat insulinoma β-cells (CRL-11605, ATCC, Manassas, VA, USA) were seeded at 3 x 10^5^ cell/cm^2^ in 6-well plates and grown in ATCC-formulated RPMI 1640 medium (Invitrogen) with 10% fetal bovine serum (FBS) (Invitrogen) and antibiotic-antimycotic (Sigma) for 24 h. Afterwards, cells were serum-starved for 24 h and then stimulated with palmitic acid (200 μmol/L) (P0500, Sigma) diluted in DMEM 5% BSA in the presence or absence of guanylin (H-2996, Bachem, Bubendorf, Switzerland) (10 nmol/L) or uroguanylin (H-2166, Bachem) (10 nmol/L) for 24 h. The concentrations of guanylin peptides and palmitic acid to carry out the experiments were chosen on the basis of previous studies performed in our laboratory (28, 44). Culture media were collected and centrifuged at 1,000 *g* for 10 min at 4 °C. Insulin release to culture media was determined by ELISA (#90060, Crystal Chem, Inc.). Intracellular triacylglycerol (TG) content was measured by enzymatic methods using the Infinity™ Triglycerides Liquid Stable Reagent (Thermo Scientific, Melbourne, Australia), as earlier described (45).

### 2.5 Real-time PCR

Transcript levels for guanylate cyclase activator 2A (*Guca2a*), guanylate cyclase activator 2B (*Guca2b*), guanylate cyclase 2C (*Gucy2c*), peroxisome proliferator-activator receptor γ (*Pparg*), sterol regulatory element binding factor 1c (*Srebf1*), monoacylglycerol *O*-acyltransferase 2 (*Mogat2*), diacylglycerol *O*-acyltransferase 1 (*Dgat1*), insulin 1 (*Ins1*) and 2 (*Ins2*), glucagon (*Gcg*), pancreatic and duodenal homeobox 1 (*Pdx1*), neurogenin 3 (*Neurog3*), Wnt family member 4 (*Wnt4*), C-C motif chemokine ligand 2 (*Ccl2*) and tumor necrosis factor α (*Tnf*) were quantified by real-time PCR (7300 Real-Time PCR System; Applied Biosystems, Foster City, CA, USA). Primers and probes (**Supplemental Table 1**) were designed using the software Primer Express 2.0 (Applied Biosystems) and were synthesized by Sigma. BLAST analysis (https://blast.ncbi.nlm.nih.gov/Blast.cgi) was applied to evaluate the specificity of the sequences of primers and probes. The thermal cycling conditions included an initial denaturation at 95°C for 10 min followed by 45 cycles at 95 °C for 15 s and extension at 59°C for 1 min, using the TaqMan^®^ Universal PCR Master Mix (Applied Biosystems). The primer and probe concentrations were 300 and 200 nmol/L, respectively. All reactions were performed in duplicate and expression levels were normalized to *18S* rRNA (Applied Biosystems) for each sample, and relative quantification was calculated using the 2^-ΔΔCt^ formula (46).

### 2.6 Western-blot

Pancreas protein samples (50 µg) were denatured and separated by SDS-PAGE in Any kD™ Mini-PROTEAN^®^ TGX™ Precast Gels (Bio-Rad) at 200 V for up to 45 min and transferred to polyvinylidene difluoride (PVDF) membranes (Bio-Rad) using the Trans-Blot Turbo transfer system (Bio Rad) at 2.5 A for 10 min. Blots were blocked in Tris-buffered saline (10 mmol/L Tris-HCl, 150 mmol/L NaCl, pH 8.00) with 0.05% Tween 20 (TBS-T) containing 5% non-fat dry milk for 1 h at room temperature (RT). Blots were then incubated overnight at 4°C with specific antibodies against GUCA2A, GUCA2B, GUCY2C and β-actin diluted in blocking solution (**Supplemental Table 2**). The antigen-antibody complexes were visualized using horseradish peroxidase-conjugated anti-rabbit or anti-mouse IgG antibodies (diluted 1:5,000 in blocking solution) and the Pierce™ ECL Plus Western-blotting Substrate (Thermo Scientific) using the ChemiDoc MP imaging system (Bio-Rad). Band density was quantified using Image Lab 6.0 software (Bio-Rad) and values were normalized to β-actin.

### 2.7 Immunohistochemistry of GUCY2C

Formalin-fixed paraffin-embedded rat pancreas sections (4 µm) were used to detect GUCY2C by the indirect immunoperoxidase method (25). Slides were deparaffinized in xylene and rehydrated in graded ethanol solutions. Endogenous peroxidase activity was blocked by incubation with 3% H_2_O_2_ (Sigma) in absolute methanol for 10 min at RT, followed by washing in absolute ethanol. Sections were heated in a microwave oven for 20 min at 800 W and 400 W in a 10 mmol/L sodium citrate buffer (pH 6.00) for antigen retrieval. Nonspecific binding was blocked with 1% goat serum (Sigma) diluted in Tris-buffer saline (TBS) (50 mmol/L Tris, 0.5 mol/L NaCl; pH 7.36) for 1 h at RT. Slides were incubated overnight at 4°C with rabbit polyclonal anti-GUCY2C (HPA037655, Sigma) diluted 1:100 in TBS. After washing the slides in TBS for three times, a pure DAKO Real™ EnVision™ anti-rabbit/mouse HRP polymer (K5007; Dako, Golstrup, Denmark) was added for 1 h at RT. Peroxidase reaction was visualized using a 0.5 mg/mL diaminobenzidine (DAB)/0.03% H_2_O_2_ solution diluted in 50 mmol/L Tris-HCl, pH 7.36, and Harris hematoxylin solution (Sigma) as counterstaining. Negative control slides without primary antibody were included to assess non-specific staining.

### 2.8 Imaging and quantification of β-cell area and number

An immunohistochemistry of insulin was performed to identify β-cells by using guinea pig polyclonal anti-insulin antibody (A0564, Dako) diluted 1:100 in rat pancreas sections (4 µm), as previously described (13). Images of insulin-positive pancreatic β-cells in Langerhans islets were captured in all fields from each animal at a total magnification of 200X, and their area and number were assessed using the software AxioVision Release 4.6.3 (Zeiss, Göttingen, Germany).

### 2.9 Measurement of pancreatic intracellular triacylglycerols

Pancreatic TG content was measured by enzymatic methods using the Infinity™ Triglycerides Liquid Stable Reagent (Thermo Scientific), as previously described (13). Briefly, pancreas samples were homogenized and diluted in saline at a final concentration of 50 mg/mL. Pancreas homogenates were diluted (1:1) in 1% deoxycholate (Sigma) and incubated at 37°C for 5 min. Afterwards, samples were diluted 1:100 in the Infinity™ Triglycerides Liquid Stable Reagent (Thermo Scientific) and incubated for 30 min at 37°C. The resulting dye was measured based on its absorbance at 550 nm. Concentrations were determined compared with a standard curve of TG (Infinity™ Triglycerides Standard, Thermo Scientific). The protein content of the preparations was measured by the Bradford assay. All assays were performed in duplicate.

### 2.10 Determination of macrophage infiltration in rat pancreas

The immunodetection of the pan-macrophage marker CD68 in rat pancreas sections was performed by the indirect immunoperoxidase method, using a mouse monoclonal anti-CD68 (ab31360, abcam) antibodies, as previously described (44). CD68-positive cells were counted in a blind study under high magnification (400X) using a light microscope (Axiovert 40 CFL, Zeiss).

### 2.11 Statistical analysis

Data are expressed as the mean ± SEM. The normality of the variables of the study was assessed by the Kolmogorov-Smirnov’s and Shapiro-Wilk’s test. Statistical differences between mean values were analyzed using Student’s t-test, χ^2^ test, one-way ANOVA followed by Tukey’s *post-hoc* test or two-way ANOVA, where appropriate. Pearson’s correlation coefficients (*r*) were used to analyze the association between variables. A *P* value <0.05 was considered statistically significant. Statistical analyses were performed using the GraphPad Prism version 6.0 and SPSS 15.0.

## 3 Results

### 3.1 Increased plasma guanylin peptides and incretin GLP-1 following sleeve gastrectomy in rats with diet induced obesity

**Figure 1:**
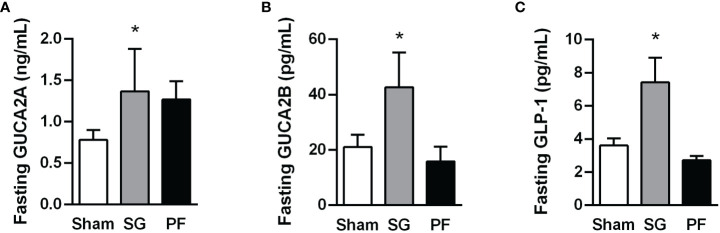
Increase in plasma guanylin peptides and GLP-1 levels following sleeve gastrectomy in experimental diet-induced obesity. Fasting GUCA2A **(A)**, GUCA2B **(B)** and GLP-1 **(C)** concentrations one month after surgical or dietary interventions. Statistical differences were analyzed using a one-way ANOVA followed by a Tukey’s *post hoc* test. *P<0.05 vs sham-operated rats.

As expected, HFD feeding increased adiposity and induced insulin resistance in experimental animals (**Supplemental Figure 1**). After sleeve gastrectomy, rats with diet-induced obesity exhibited reduced markers of adiposity (body weight, whole-body adiposity and the adipokine leptin) as well as an improved insulin sensitivity (**Supplemental Figure 2**). A significant increase in circulating GUCA2A and GUCA2B (both P<0.05) was observed following sleeve gastrectomy compared to other groups (**Figures 1A, B**). Interestingly, plasma GUCA2A levels, but not GUCA2B, were negatively correlated with circulating insulin (r=-0.52, *P*=0.007) and HOMA index (r=-0.47, *P*=0.017). A rise in plasma concentrations of GLP-1, a gut hormone involved in the improvement of insulin secretion from β-cells after bariatric surgery, was also observed after sleeve gastrectomy (*P*<0.05) (**Figure 1C**).

### 3.2 Expression of the guanylin system in rat pancreas in obesity and after weight loss achieved by sleeve gastrectomy

**Figure 2:**
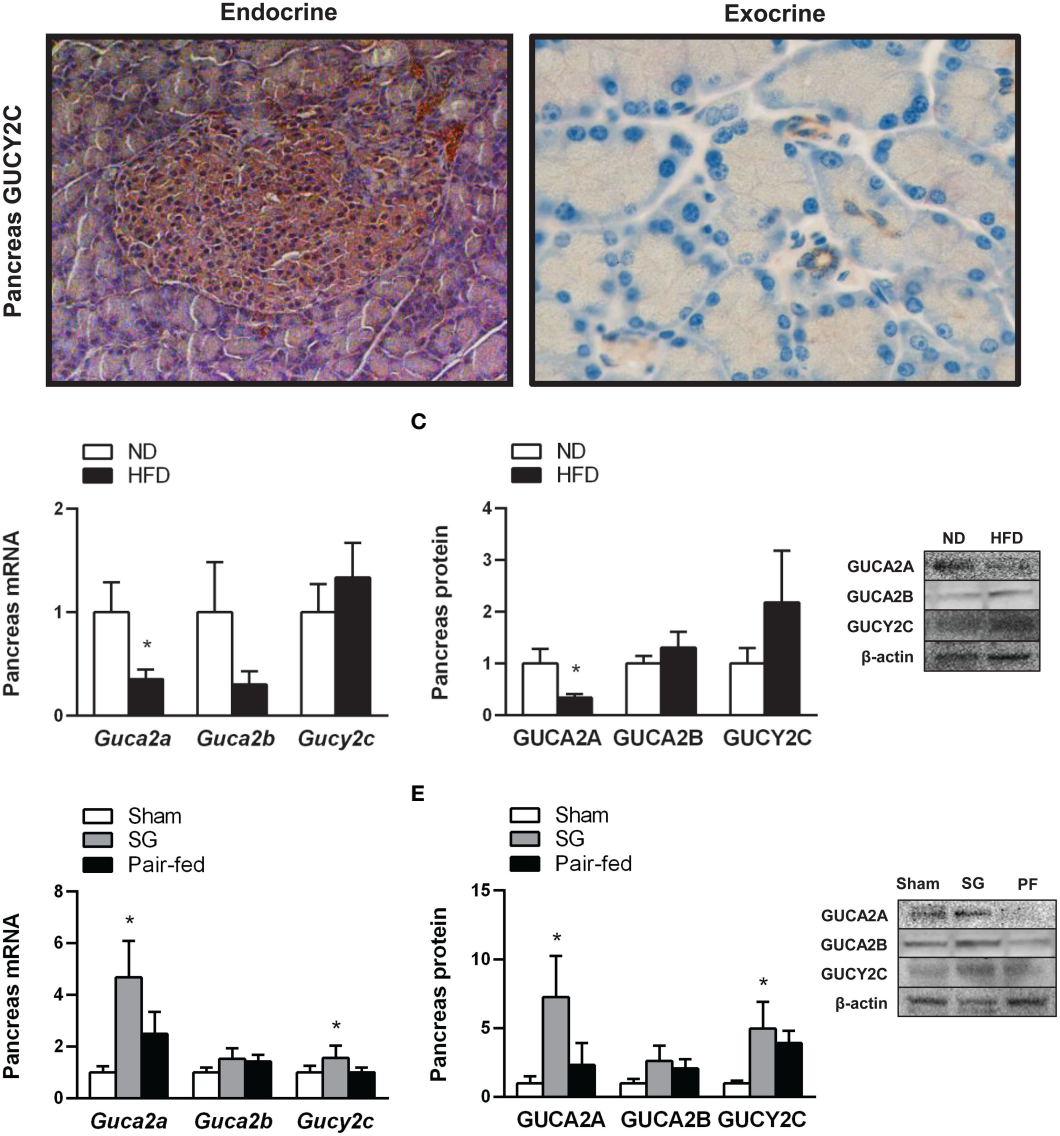
Effect of obesity and weigh loss achieved by sleeve gastrectomy on the expression of the guanylin system in rat pancreas. **(A)** Cellular localization of the receptor of guanylin peptides, GUCY2C, in the islets of Langerhans (*left panel*, magnification 200X) as well as in inter- and intralobular pancreatic ducts (*right panel*, magnification 400X) in rat pancreas. Bar graphs illustrate the impact of weight gain induced by HFD feeding **(B, C)** and weight loss achieved by sleeve gastrectomy **(D, E)** on pancreatic mRNA and protein of GUCA2A, GUCA2B and their receptor GUCY2C. The gene and protein expression in lean control rats fed a ND or in the sham-operated group was assumed to be 1. Representative blots are shown. Statistical differences were analyzed using Student’s *t*-test or one-way ANOVA followed by a Tukey’s *post hoc* test, where appropriate. *P<0.05 *vs* lean or sham-operated rats.

To confirm whether the rat pancreas constitutes a target for the metabolic effects of the guanylin peptides, the presence of GUCY2C in the pancreas from the experimental animals was evaluated by immunohistochemistry. A positive staining for GUCY2C was observed in the islets of Langerhans as well as in pancreatic duct cells (**Figure 2A**), highlighting its involvement in the function of exocrine and endocrine pancreas. We next evaluated the impact of obesity and weight loss achieved by sleeve gastrectomy in the guanylin system in the pancreas. Real-time PCR analyses confirmed a previous study (37) showing that proguanylin is highly expressed in the pancreas, whereas transcriptional levels of prouroguanylin are almost negligible in all samples compared to those of proguanylin (*Guca2a* 1.00 ± 0.00 *vs Guca2b* 0.21 ± 0.07 A.U., *P*<0.0001). A decrease in GUCA2A mRNA and protein expression (both *P*<0.05) was found in the pancreas of rats with diet-induced obesity, whereas GUCA2B and GUCY2C gene and protein differences fell out of statistical significance (**Figures 2B, C**). One month after sleeve gastrectomy, an upregulation in pancreas RNA and protein expression of GUCA2A and its receptor GUCY2C was observed (**Figures 2D, E**). Importantly, univariate analyses revealed a negative association between pancreatic protein levels of GUCA2A (r=-0.30, *P*<0.05) and GUCA2B (r=-0.32, *P*<0.05) with insulinemia, supporting the active role of both peptides in β-cell insulin secretion.

### 3.3 Sleeve gastrectomy ameliorated pancreas steatosis and inflammation in rats with diet-induced obesity

**Figure 3:**
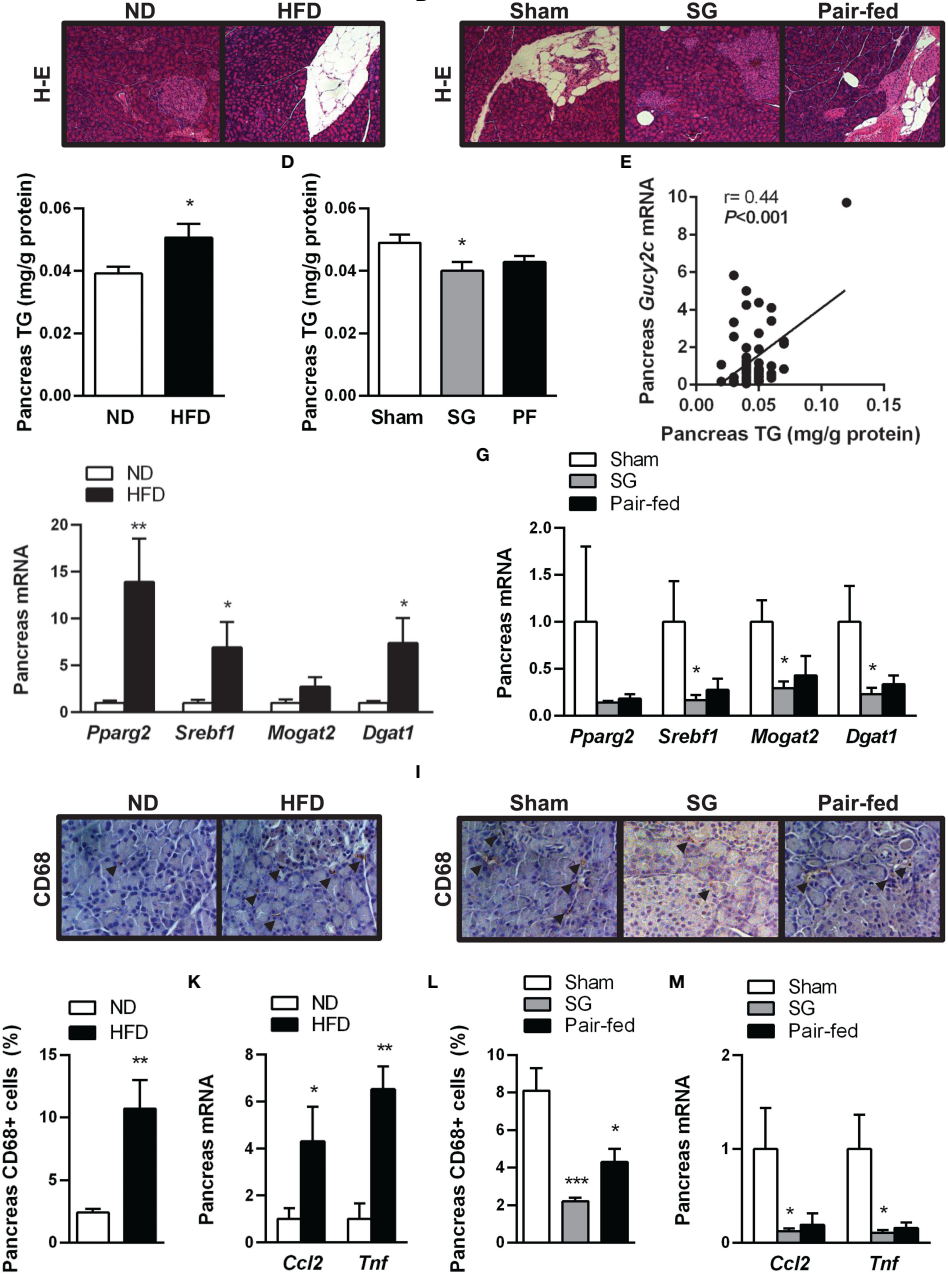
Improvement of obesity-associated pancreas steatosis and inflammation after sleeve gastrectomy. Hematoxylin-eosin-stained pancreas sections showing pancreas steatosis in **(A)** rats with diet-induced obesity and **(B)** after surgical and dietary interventions (magnification 100X). Impact of diet-induced obesity as well as weight loss achieved by sleeve gastrectomy on pancreatic triacylglycerol (TG) content **(C, D)** and transcript levels of lipogenic transcription factors and enzymes **(F, G)**. **(E)** Correlation between pancreatic *Gucy2c* mRNA and pancreatic steatosis. Representative images showing the brown staining for pan-macrophage marker CD68 in sections of the pancreas of **(H)** rats with diet-induced obesity and **(I)** after surgical and dietary interventions (magnification 200X); arrows indicate CD68^+^ cells. Bar graphs show the effect of diet-induced obesity and weight loss achieved after sleeve gastrectomy on macrophage infiltration **(J, L)** and gene expression of proinflammatory factors **(K, M)** in the pancreas. *P< 0.05; **P<0.01; ***P<0.001 *vs* lean or sham-operated rats.

HFD feeding led to increased pancreas fat accumulation (*P*<0.05) (**Figures 3A, C**) together with an upregulation (all *P*<0.05) in factors involved in lipogenic transcription factors *Pparg* and *Srebf1* and downstream enzyme *Dgat1* (**Figure 3F**). One month after sleeve gastrectomy, rats with diet-induced obesity exhibited a reduction in pancreatic steatosis (**Figures 3B, D**) as well as a downregulation of lipogenic transcription factors and enzymes (**Figure 3G**). Interestingly, a positive correlation of GUCY2C with pancreas fat accumulation was found (**Figure 3E**), suggesting a potential involvement of guanylin peptides in the regulation of pancreatic lipid metabolism.

During the onset of obesity, increased adiposity is associated with chronic low-grade inflammation. In this regard, not only subcutaneous and visceral adipocytes, but also pancreatic adipocytes secrete cytokines, chemokines and chemoattractants that can predispose to pancreatic inflammation (7). In line with this observation, our histological analyses revealed an increased CD68^+^ macrophage accumulation in both endocrine and exocrine pancreata from rats with diet-induced obesity (**Figures 3H, J**). In accordance with inflammatory cell infiltration, an upregulation (*P*<0.05) in transcript levels of chemokine MCP-1 (*Ccl2*) and cytokine TNF-α (*Tnf*) was observed in the pancreas of rats with diet-induced obesity (**Figure 3K**). Sleeve gastrectomy markedly reduced (*P*<0.001) CD68^+^ cells (**Figures 3I, L**) and the mRNA expression of proinflammatory factors *Ccl2* and *Tnf* (both *P*<0.05) (**Figure 3M**) in rat pancreas. The reduction in pancreatic fat and inflammatory cell content was more evident in the group of rats submitted to sleeve gastrectomy than in that subjected to pair feeding, suggesting that the post-surgical improvement of pancreas steatosis and inflammation is beyond caloric restriction.

### 3.4 Sleeve gastrectomy improved β-cell function and identity in rats with diet-induced obesity

**Figure 4:**
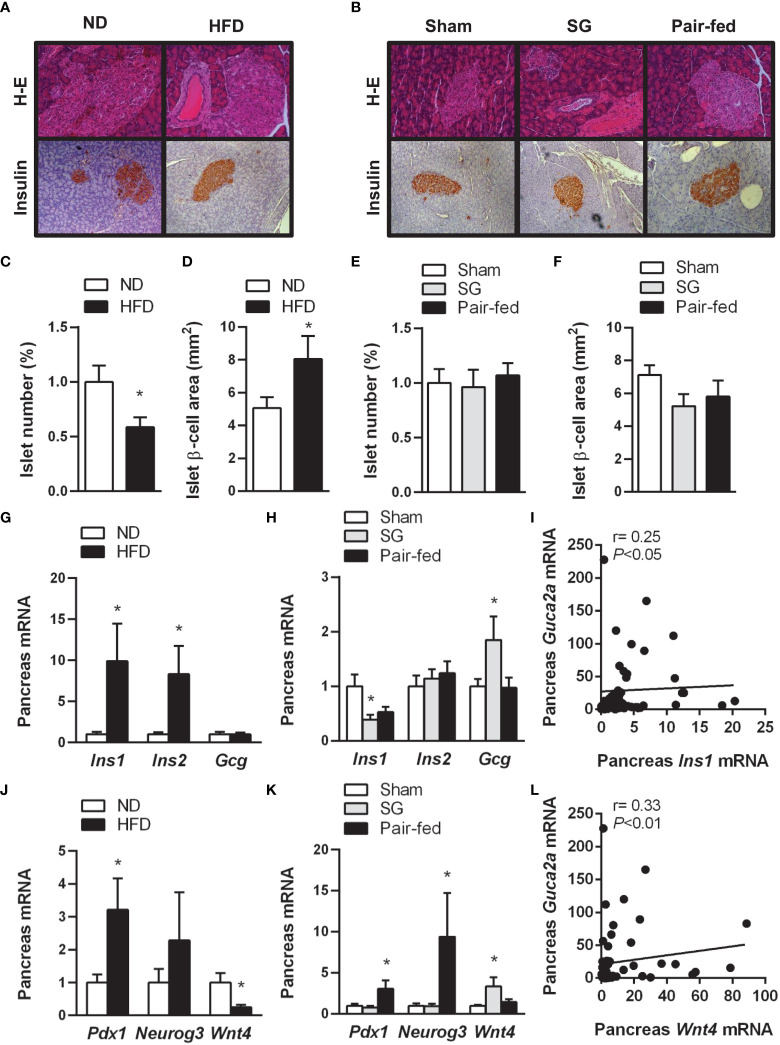
Amelioration of pancreatic β-cell function and identity after sleeve gastrectomy. Representative images of sections of pancreas from **(A)** rats with diet-induced obesity and **(B)** after surgical and dietary interventions stained with H-E (*upper panels*) or marked with antibodies against insulin (*bottom panels*) (magnification 200X). Bar graphs illustrate the impact of obesity and weight loss achieved by sleeve gastrectomy in islet number **(C, E)**, β-cell area **(D, F)** as well as gene expression involved in the pancreas endocrine function **(G, H)** and β-cell identity **(J, K)**. Correlation between pancreatic transcript levels of *Guca2a* with mRNA expression of *Ins1* **(I)** and *Wnt4* **(L)**. *P<0.05 *vs* lean control rats or sham-operated group.

To gain further insight into the impact of obesity on β-cell dysfunction (47), we investigated the β-cell mass and the expression of factors involved in α- and β-cell endocrine function (*Ins1, Ins2* and *Gcg*) in pancreata from the experimental animals. Representative images of islets and β-cell area stained with antibodies against insulin in the pancreas are illustrated in **Figures 4A, B**. Obesity was associated with a reduction (*P*<0.05) of islet density (**Figure 4C**), but with more than 1.5-fold increase in β-cell mass (*P*<0.05) (**Figure 4D**) accompanied by increased insulin-1 and insulin-2 transcripts (**Figure 4G**). Rats submitted to sleeve gastrectomy did not change the total number of islets or the β-cell area (**Figures 4E, F**). Nonetheless, pancreatic endocrine function was improved after sleeve gastrectomy, as evidenced by a decrease in *Ins1* mRNA and an upregulation of mRNA levels of glucagon (**Figure 4H**).

The molecular identity and unique functionality of β-cells are defined by the expression of specific transcription factors (e.g. *Pdx1, Neurog3, Mafa* or *Pax6*) that govern pancreatic islet morphogenesis. Interestingly, under conditions of metabolic stress, loss of mature β-cell identity is critical for β-cell failure (48, 49). Thus, we evaluated the expression of crucial factors involved in β-cell reprogramming, such as *Pdx1, Neurog3* and the recently discovered *Wnt4*, a Wnt ligand that fine-tune regulates β-cell proliferation at low levels and β-cell maturation at high levels (50). The pancreata of rats with diet-induced obesity showed a high expression of *Pdx1* (*P*<0.05) together with low transcript levels of *Wnt4* (*P*<0.05), without changes in *Neurog3* (**Figure 4J**). Interestingly, rats with diet-induced obesity in the pair-fed group maintained high levels of *Pdx1* and *Neurog3* (**Figure 4K**), pointing to an increased formation of insulin-secreting β-cells. By contrast, after sleeve gastrectomy, no changes were observed in the expression of *Pdx1* and *Neurog3*. Interestingly, *Wnt4* mRNA expression was upregulated after sleeve gastrectomy, suggesting that the changes in β-cell function after surgery are more related to β-cell maturation and functionality rather than to an increased β-cell proliferation. A positive correlation of *Guca2a* transcript levels with *Ins1* and *Wnt4* was detected (**Figures 4I, L**).

### 3.5 Protective effect of guanylin peptides against palmitate-β-cell steatosis and insulin secretion

**Figure 5:**
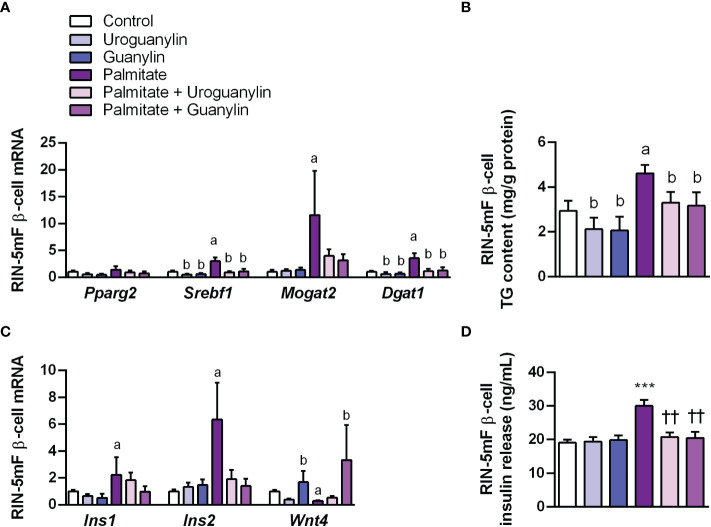
Effect of guanylin peptides on palmitate-induced steatosis, insulin secretion and loss of identity in rat RIN-5mF β-cells. **(A)** mRNA expression of lipogenic transcription factors and enzymes **(B)** intracellular triacylglycerol (TG) content in rat RIN-5mF β-cells co-treated with palmitate and guanylin peptides for 24 h. Transcript levels of genes involved in insulin synthesis and β-cell morphogenesis **(C)** as well as insulin secreted to the culture media **(D)** Statistical differences were analyzed by two-way ANOVA or one-way ANOVA followed by Tukey´s *post-hoc* test, in case of interaction between factors. ***P<0.001 *vs* unstimulated cells; ††*P*<0.01 *vs* palmitate-treated RIN-5mF cells.; †*P*<0.05; ††*P*<0.01 *vs* palmitate-treated RIN-5mF cells; ^a^ *P*<0.05 effect of palmitate; ^b^ *P*<0.05 effect of uroguanylin or guanylin.

Palmitate (16:0) is the most common saturated fatty acid found in the circulation and its excessive dietary consumption is associated with obesity and its associated comorbidities, including insulin resistance and type 2 diabetes, among others (51). In the pancreas, palmitate enhances glucose-stimulated insulin secretion from isolated islets, with chronic exposure ultimately leading to β-cell dysfunction (11, 52). Thus, the direct effect of guanylin peptides against β-cell lipotoxicity was tested in rat RIN-5mF β-cells exposed to palmitate (200 μmol/L), in the absence or presence of uroguanylin or guanylin (both 10 nmol/L) for 24 h. As expected, palmitate treatment upregulated (all *P*<0.05) gene expression levels of the lipogenic factors *Srebf1, Mogat2* and *Dgat1* (**Figure 5A**) at the same time as it increased (*P*<0.01) intracellular TG content (**Figure 5B**). Both uroguanylin and guanylin downregulated basal and palmitate-induced transcription of *Srebf1* and *Dgat1* as well as steatosis in RIN-5mF β-cells (all *P*<0.05) (**Figures 5A, B**). Moreover, palmitate treatment for 24 h increased (both *P<*0.05) *Ins1* and *Ins2* mRNA (**Figure 5C**) as well as insulin secretion (*P*<0.001) (**Figure 5D**) from RIN-5 mF β-cells. Both uroguanylin and guanylin reduced palmitate-induced transcription of *Ins1* and *Ins2* as well as insulin secretion (*P*<0.01) in RIN-5 mF β-cells (**Figures 5C, D**). No effect of both guanylin peptides on basal insulin secretion were observed. Interestingly, palmitate-treated β-cells exhibited a dramatic downregulation (*P*<0.001) of *Wnt4* mRNA levels in β-cells, while in the presence of guanylin, but not uroguanylin, *Wnt4* transcript levels were markedly increased (*P*<0.001) (**Figure 5C**).

## 4 Discussion

Pancreatic steatosis is associated with insulin resistance and β-cell dysfunction (13, 53) and accelerates the process of inflammation in acute pancreatitis (54). Increased exposure to exogenous long-chain fatty acids, such as palmitate (C16:0), oleate (C18:1n9) and stearate (C18:0), differentially impact β-cells over differing periods of time. Short-term exposure to palmitate, the most abundant circulating saturated fatty acid, is associated with post-prandial insulin secretion (55), while a decline in islet β-cell function and mass is observed after its prolonged exposure (11, 52). In line with these observations, we herein described an increased steatosis, proinsulin synthesis and insulin secretion in β-cells exposed to palmitate *in vitro*, as well as an increased pancreas fat accumulation and inflammation together with a marked decrease in islet number in rats fed a HFD. Reductions in the number of insulin-secreting β-cells in pancreatic islets with the simultaneous expansion of glucagon-secreting α-cells (56) as well as insulin hypersecretion in the absence of insulin resistance (57) often precede the onset of type 2 diabetes. In this regard, a direct contribution of an altered expression of transcription factors involved in the maintenance of β-cell identity (e.g. PDX1, NKX6.1, MAFA or NEUROG3, among others) has been proposed to explain β-cell failure under pathological conditions of hyperglycemia and lipotoxicity (48, 58). Our data revealed a significant increase in PDX1 and a decrease in WNT4 expression in pancreata from rats with diet-induced obesity, which might be involved in the compensatory β cell mass expansion in response to obesity-associated insulin resistance through their roles in cell proliferation and function (50, 58).

Bariatric surgery has a proven effect on type 2 diabetes improvement (1). Several studies have shown a reduction in pancreatic fat accumulation following bariatric surgery, an effect that might improve glucose homeostasis (13, 59, 60). Accordingly, an amelioration of pancreas steatosis together with a downregulation of the lipogenic genes *Srebf1, Mogat2* and *Dgat1* was observed following sleeve gastrectomy. Moreover, our data support that this bariatric surgical procedure diminished macrophage infiltration in the pancreas. A limitation of this study is that we have used the pan-macrophage marker CD68 to detect macrophages, which does not distinguish M1 and M2 phenotypes. Nonetheless, recent in-depth analyses of immune cell populations using single-cell technologies have shown the full heterogeneity and plasticity of the immune cell compartment in obesity, supporting the notion that macrophages are not strongly polarized toward the classical M1 and M2 macrophage states (61). Instead, macrophage subpopulations constitute a continuum of cell states, where some are more proinflammatory and others are more metabolically active. In this sense, we herein show that sleeve gastrectomy downregulated the mRNA synthesis of the proinflammatory factors MCP-1 and TNF-α in pancreata of the rats with diet-induced obesity, suggesting that this bariatric procedure might contribute to the polarization of macrophages towards a less proinflammatory state. Sleeve gastrectomy also increased insulin sensitivity, evidenced by lower basal glucose levels and AUC during the OGTT and IPITT as well as lower HOMA index, which is in accordance with previous reports (25, 62–65). A post-surgical improvement in the endocrine function of the pancreas was found, as shown by the decrease in proinsulin synthesis and increase in glucagon synthesis, without changes in islet area or number. Interestingly, rats with pair-feeding only exhibited a modest decrease in insulinemia and proinsulin synthesis, suggesting that the beneficial effects of sleeve gastrectomy on the pancreas endocrine function are beyond caloric restriction. In line with this observation, an upregulation of *Pdx1* and *Neurog3* was observed in the pair-fed group, suggesting a compensatory β cell mass expansion under caloric restriction that is no longer needed following sleeve gastrectomy. By contrast, the high *Wnt4* transcripts in pancreata after sleeve gastrectomy might reflect an improved β-cell maturation and adaptive insulin response (50). Together, sleeve gastrectomy is an effective procedure to reduce pancreas steatosis and inflammation, contributing to an improved β-cell insulin secretion and insulin sensitivity.

It is well accepted that several gut hormones, such as glucagon-like peptide 1 (GLP-1), gastric inhibitory peptide (GIP) or ghrelin are involved in the improvement of β-cell function after bariatric surgery (13, 66, 67). Interestingly, circulating GUCA2A and GUCA2B levels are also increased after sleeve gastrectomy and Roux-en-Y gastric bypass (25, 28, 68). In line with these observations, an increase in plasma GUCA2A, GUCA2B and GLP-1 levels was observed following sleeve gastrectomy. Although the intestine represents the main production site of guanylin peptides, the pancreas expresses the complete guanylin system (37). Thus, the potential beneficial effect of guanylin peptides on pancreas steatosis and insulin secretion was investigated *in vivo* and in an *in vitro* model of NAFP, namely RIN-m5F β cells stimulated with palmitate. We herein show, for the first time, that obesity reduces GUCA2A mRNA and protein expression in the pancreas, and weight loss achieved by sleeve gastrectomy upregulated both GUCA2A and GUCY2C expression. Interestingly, protein levels of GUCA2A and GUCA2B in the pancreas were negatively associated with insulinemia, suggesting an active role of guanylin peptides in insulin secretion. In this regard, a previous study reported that guanylin can induce a modest increase in insulin secretion in rat BRIN-BD11 β-cells (41). By contrast, our data revealed that guanylin peptides cannot modify the mRNA expression of *Ins1* and *Ins2* or the secretion of insulin in β-cells under basal state, but both guanylin and uroguanylin blunted palmitate-induced insulin secretion. In addition, guanylin treatment increased *Wnt4* transcription in β-cells under basal and lipotoxic conditions. A positive association between *Guca2a* and *Wnt4* transcripts was also observed in the pancreas of experimental animals, supporting that guanylin contributes to improved β-cell function *via* Wnt4 signalling. Importantly, both guanylin and uroguanylin decreased basal and palmitate-induced steatosis and downregulated factors involved in lipogenesis (*Srebf1, Mogat2* and *Dgat1*). Nonetheless, the involvement of other potential factors like aquaporins cannot be discarded (10, 13, 14, 69, 70).

In conclusion, we herein show, for the first time, that guanylin and uroguanylin prevent β-cell steatosis and insulin secretion under lipotoxic conditions. Obesity is associated with low pancreas expression of GUCA2A, the most abundant guanylin peptide in this endocrine organ. Importantly, weight loss achieved by sleeve gastrectomy increased pancreatic GUCA2A and its receptor GUCY2C in parallel to the reduction of pancreas fat accumulation, macrophage accumulation, proinflammatory cytokine production and improved β-cell function. These results support the notion that GUCA2A mediates, at least in part, the benefits of bariatric surgery on pancreas steatosis and function.

## Data availability statement

The original contributions presented in the study are included in the article/**Supplementary Materials**, further inquiries can be directed to the corresponding authors.

## Ethics statement

The animal study was reviewed and approved by Ethical Committee for Animal Experimentation of the University of Navarra.

## Author contributions

AR and GF contributed to conception and design of the study. AR wrote the first draft of the manuscript. AO, SB, MM, JC, VV, RM, VC, JG-A, MB and GF collected and analyzed data, contributed to discussion and reviewed the manuscript. AR and GF are guarantors for the contents of the article and had full access to all the data in the study and take responsibility for the integrity of the data and the accuracy of the data analysis. All authors contributed to the article and approved the submitted version.
