# Supplementary material for: Effect of guanylin peptides on pancreas steatosis and function in experimental diet-induced obesity and after bariatric surgery
Source: Front Endocrinol (Lausanne). 2023 May 18;14:1185456. doi: 10.3389/fendo.2023.1185456 (PMC10233012; doi:10.3389/fendo.2023.1185456)
Supplement: Supplementary file 1 [file DataSheet_1.docx]

Supplementary Material

**
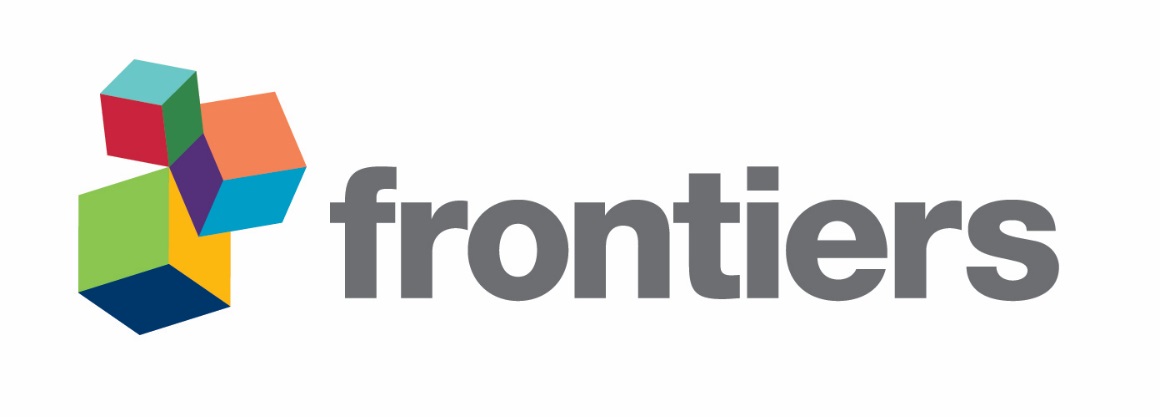
**

**Supplemental Table 1. Sequences of primers and TaqMan^®^ probes.**

| Gene  (GenBank accession no.) | | Oligonucleotide sequence (5’-3’) | | | | | Nucleotides | | |  |  |
| --- | --- | --- | --- | --- | --- | --- | --- | --- | --- | --- | --- |
| *Ccl2*  (NM_031530) | |  | | | | | |  | | | |
| Forward | | TCTTGGGACTGATGTTGTTGACA | | | | | | 105-127 | | | |
| Reverse | | catatgttctcagggagatcttgga | | | | | | 231-255 | | | |
| Taqman^®^ probe | | FAM-TCACAGAGGATACCACCCACAACAGACCA-TAMRA | | | | | | 168-196 | | | |
| Dgat1  (NM_053437.1) | | |  | |  | | |  |  |  |  |
| Forward | | CGGTCCCCAACCATCTGATAT | | | 1048-1068 | | |  |  |  |  |
| Reverse | | tttccactcatgtctcaatgctgtggca | | | 1132-1152 | | |  |  |  |  |
| Taqman^®^ probe | | FAM-TTCCACTCATGTCTCAATGCTGTGGCA-TAMRA | | | 1091-2018 | | |  |  |  |  |
| *Gcg*  (NM_012707.3) | |  | | | | | |  | | | |
| Forward | | CGCCGTGCTCAAGATTTTGT | | | | | | 298-317 | | | |
| Reverse | | ttgagaggcatgctgaaggg | | | | | | 377-396 | | | |
| Taqman^®^ probe | | FAM-CAAGAGGAACCGGAACAACATTGCCA-TAMRA | | | | | | 336-361 | | | |
| *Guca2a*  (NM_013118.1) | |  | | | | | |  | | | |
| Forward | | CTGTGCAGGATGGAGACCTTTC | | | | | | 80-101 | | | |
| Reverse | | aagtttgctctcaggcttccg | | | | | | 175-195 | | | |
| Taqman^®^ probe | | FAM-CGGGAAGTACAGGAGCCCACACTGATG-TAMRA | | | | | | 138-165 | | | |
| *Guca2b*  (NM_022284.2) | |  | | | | | |  | | | |
| Forward | | TCCCCAGCAGCAGAAAAGTG | | | | | | 183-202 | | | |
| Reverse | | cttgaggaccattgccactga | | | | | | 294-314 | | | |
| Taqman^®^ probe | | FAM-CTCCTCCCCGATGTGTGCTACA-TAMRA | | | | | | 205-226 | | | |
| *Gucy2c*  (NM_013170.1) | |  | | | | | |  | | | |
| Forward | | TCAGCCTGAAGATTGACGATGA | | | | | | 1467-1487 | | | |
| Reverse | | aaagacctcaagcactgtgatgg | | | | | | 1553-1575 | | | |
| Taqman® probe | | FAM-ACAATCCAGAGAGTGCGACAGTGCAA-TAMRA | | | | | | 1501-1527 | | | |
| Ins1  (NM_019129.3) | | | | | |  | |  | | | |
| Forward | | | | GCCCAGGCTTTTGTCAAACA | | | | 121-140 | | | |
| Reverse | | | | tcttctacacacccaagtcccg | | | | 200-221 | | | |
| Taqman^®^ probe | | | | FAM-CACCTTTGTGGTCCTCACCTGG-TAMRA | | | | 142-162 | | | |
| Ins2  (NM_019130.2) | | | |  | | | |  | | | |
| Forward | | | | CTGCTGGCCCTGCTCATC | | | | 85-102 | | | |
| Reverse | | | | tgtgtggggagcgtgga | | | | 182-198 | | | |
| Taqman^®^ probe | | | | FAM-CTGCCCAGGCTTTTGTCAAACAGCAC-TAMRA | | | | 119-144 | | | |
| Mogat2  (NM_001109436.2) | |  | | | | | |  | | | |
| Forward | | TCCCTGTCTCTTTGGTCAAGACA | | | | | | 289-311 | | | |
| Reverse | | TTCTTAACCTGTGCACTGAAAGCA | | | | | | 382-405 | | | |
| Taqman^®^ probe | | FAM-CGGAACTACATCGCAGGCTTTCACCC-TAMRA | | | | | | 330-355 | | | |
| Neurog3  (NM_021700.2) | |  | | | | | |  | | | |
| Forward | | CCGGATGACGCCAAACTTAC | | | | | | 790-809 | | | |
| Reverse | | GCATAGCGGACCACAGCTTC | | | | | | 869-888 | | | |
| Taqman^®^ probe | | FAM-CCTGCGCTTCGCCCACAACTACATT-TAMRA | | | | | | 822-846 | | | |
| Pdx1  (NM_022852.4) | |  | | | | | |  | | | |
| Forward | | CCGCGTTCATCTCCCTTTC | | | | | | 464-482 | | | |
| Reverse | | CATACGCAGCAGAACCGGA | | | | | | 538-556 | | | |
| Taqman^®^ probe | | FAM-TGGAAAAGCCAGTGGGCAGGAGGT-TAMRA | | | | | | 513-536 | | | |
| Pparg  (NM_013124) | |  | | | | | |  | | | |
| Forward | | CTGACCCAATGGTTGCTGATTAC | | | | | | 257-279 | | | |
| Reverse | | CCTGTTGTAGAGTTGGGTTTTTTCA | | | | | | 351-375 | | | |
| Taqman^®^ probe | | FAM-TGAAGCTCCAAGAATACCAAAGTGCG-TAMRA | | | | | | 290-315 | | | |
| Srebf1  (NM_001276707.1) | |  | | | | | |  | | | |
| Forward | | ATGCGGCTGTCGTCTACCAT | | | | | | 2050-2069 | | | |
| Reverse | | AGTGTGCAGGAGATGCTATATCCAT | | | | | | 2158-2182 | | | |
| Taqman^®^ probe | | FAM-CATGCCATGGGCAAGTACACAGGAGG-TAMRA | | | | | | 2085-2110 | | | |
| Tnf  (NM_012675.3) | |  | | | | | |  | | | |
| Forward | | CCAAATGGGCTCCCTCTCAT | | | | | | 337-356 | | | |
| Reverse | | tagcaaaccaccaagcggag | | | | | | 431-450 | | | |
| Taqman^®^ probe | | FAM-TTCCATGGCCCAGACCCTCACACTC-TAMRA | | | | | | 360-384 | | | |
| Wnt4  (NM_053402.2) | |  | | | | | |  | | | |
| Forward | | GGTGTGGCCTTTGCAGTGA | | | | | | 360-378 | | | |
| Reverse | | TATGGCGTAGCCTTCTCACAGTC | | | | | | 486-508 | | | |
| Taqman^®^ probe | | FAM-AGGGTTTCCAGTGGTCAGGATGCTCG-TAMRA | | | | | | 448-473 | | | |

*Ccl2,* C-C motif chemokine ligand 2; *Dgat1,* diacylglycerol *O*-acyltransferase 1; *Gcg*, glucagon; *Guca2a,* guanylate cyclase activator 2A*; Guca2b,* guanylate cyclase activator 2B; *Gucy2c,* guanylate cyclase 2C; *Ins1*, insulin 1; *Ins2*, insulin 2; *Mogat2,* monoacylglycerol *O*-acyltransferase 2; *Neurog3,* neurogenin 3; *Pdx1*, pancreatic and duodenal homeobox 1; *Pparg,* peroxisome proliferator-activator receptor γ; *Srebf1*, sterol regulatory element binding factor 1c; *Tnf*, tumor necrosis factor α; *Wnt4*, Wnt family member 4.

**Supplemental Table 2. Primary antibodies used for Western-blot.**

| Protein target | | Manufacturer (catalog number) | Species reactivity | Dilution |
| --- | --- | --- | --- | --- |
| GUCA2A | Abnova (pab21024) | | Rabbit polyclonal | 1:1,000 |
| GUCA2B | novus biologicals (h00002981-d01p) | | Rabbit polyclonal | 1:1,000 |
| GUCY2C | sigma (hpa037655) | | Rabbit polyclonal | 1:1,000 |
| β-actin | sigma (a5441) | | Mouse monoclonal | 1:5,000 |

GUCA2A, proguanylin; GUCA2B, prouroguanylin; GUCY2C, guanylate cyclase 2C.


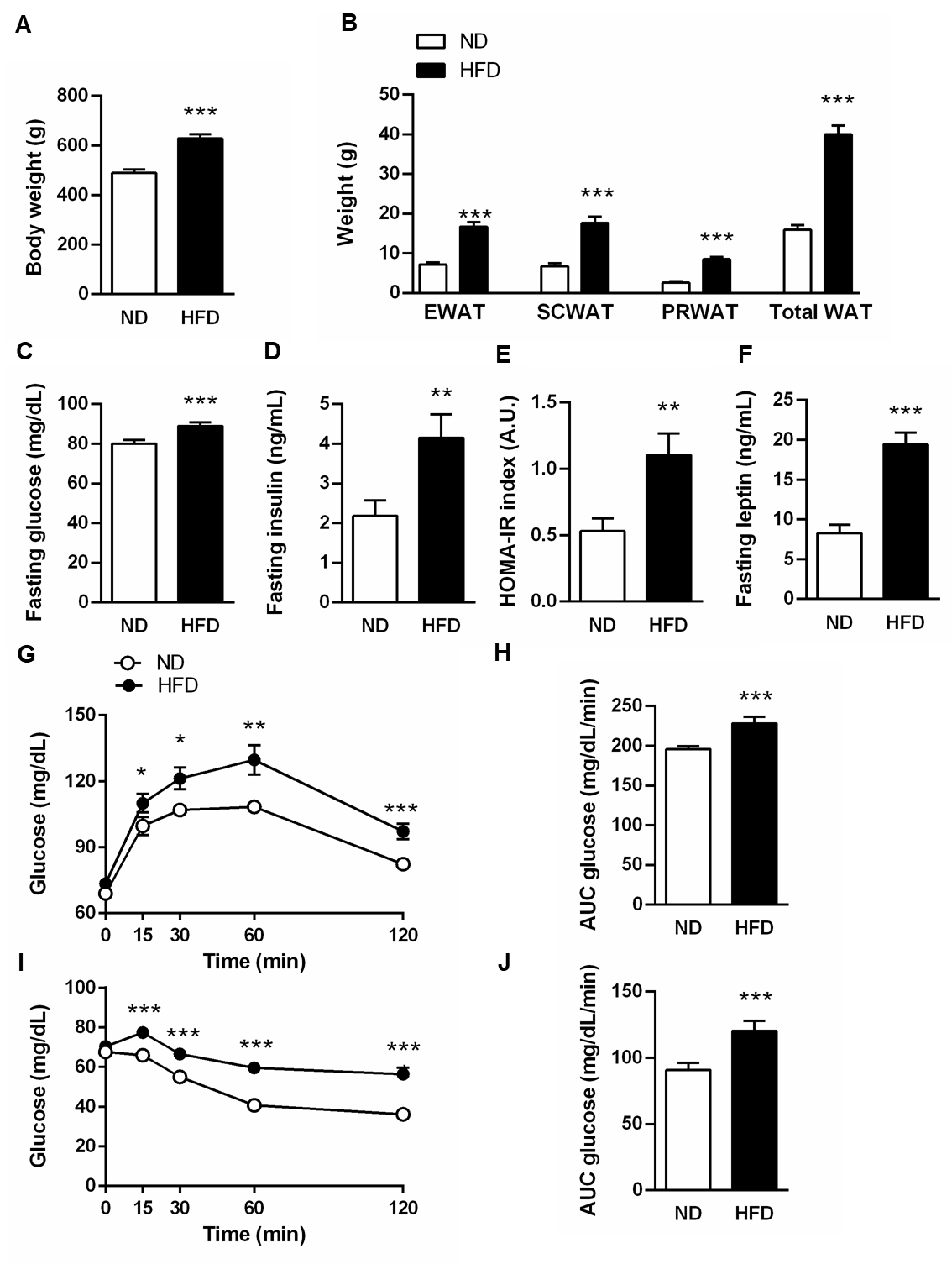


**Supplemental Fig 1**. Increased body weight, adiposity, impaired glucose tolerance and insulin resistance in rats with diet-induced obesity. Bar graphs show the (A) body weight, (B) epididymal (EWAT), subcutaneous (SCWAT), perirenal (PRWAT) fat depots and total white adiposity as well as (C) fasting glucose, (D) fasting insulin, (E) HOMA-IR index and (F) fasting leptin of rats fed a normal diet (ND) or a high-fat diet (HFD). Blood glucose levels and area under the curve (AUC) during OGTT (G and H) and IPITT (I and J). Statistical differences were analyzed by using a Student’s *t* test. **P*<0.05, ***P*<0.01, ****P*<0.001 *vs* control rats fed a ND.


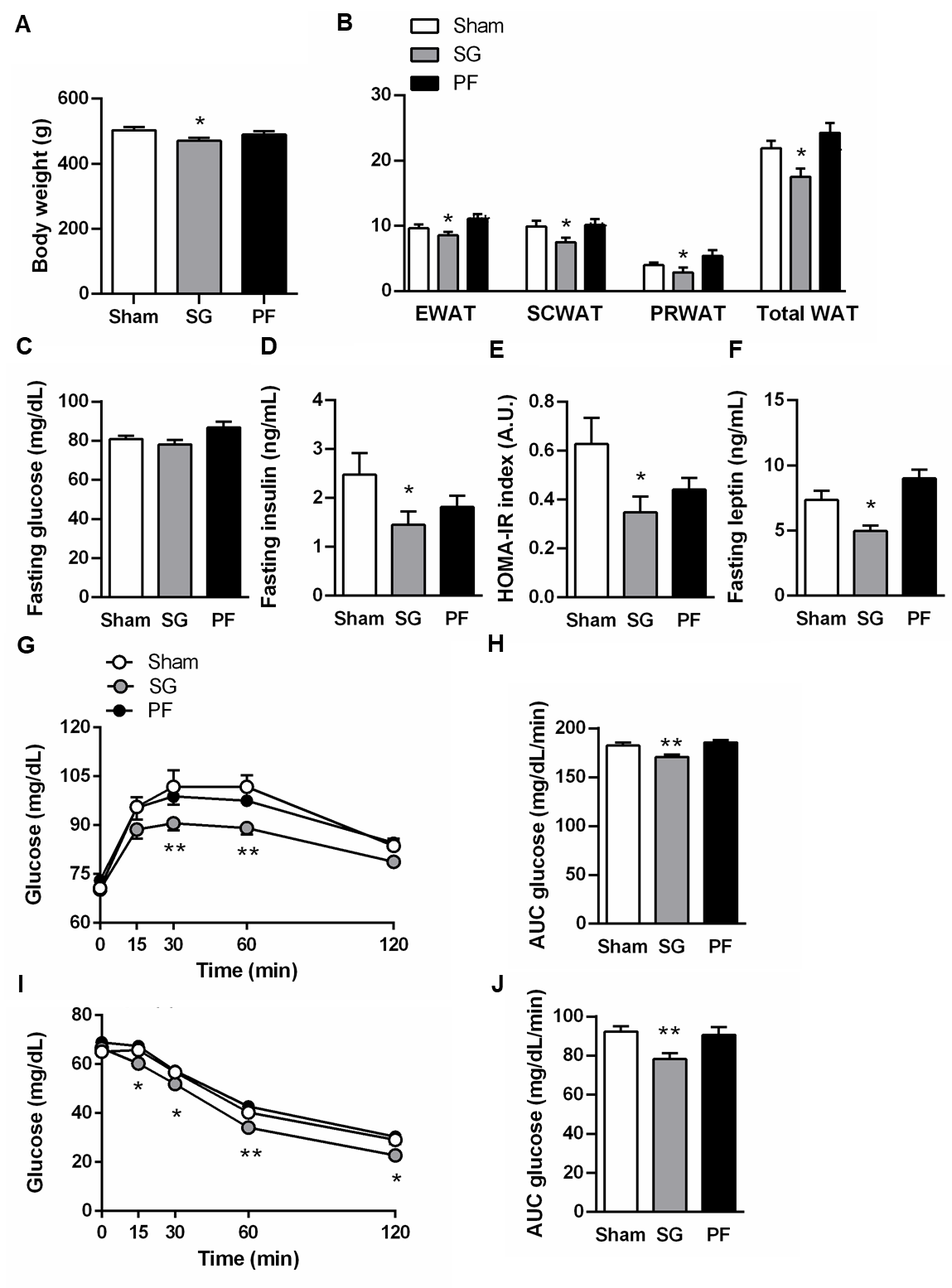


**Supplemental Fig. 2.** Bariatric surgery improved body weight, adiposity and insulin sensitivity of rats with diet-induced obesity. Bar graphs show the (A) body weight, (B) epididymal (EWAT), subcutaneous (SCWAT), perirenal (PRWAT) fat depots and total white adiposity as well as (C) fasting glucose, (D) fasting insulin, (E) HOMA-IR index and (F) fasting leptin of rats with diet-induced obesity one month after sham surgery, sleeve gastrectomy (SG) or pair-feeding (PF). Blood glucose levels and areas under the curve (AUC) during OGTT (G and H) and IPITT (I and J) are shown. Statistical differences were analyzed by one-way ANOVA followed by Tukey’s *post-hoc* test. **P*<0.05, ***P*<0.01 *vs* rats with diet-induced obesity submitted to sham surgery.
